# Starting bedtime glargine versus NPH insulin in poorly controlled type 2 diabetic patients with various hyperglycemia types (fasting type or postprandial type)

**DOI:** 10.1007/s00592-013-0505-7

**Published:** 2013-07-24

**Authors:** Markku A. Vähätalo, Jorma Viikari, Tapani Rönnemaa

**Affiliations:** 1 City of Loimaa Health Care Services, Department of Medicine, University of Turku, Turku, Finland; 2 Department of Medicine, Turku University Hospital and University of Turku, Turku, Finland

**Keywords:** Insulin treatment, Insulin analog, Hyperglycemia type, Body weight increase, Type 2 diabetes

## Abstract

Our aim was to compare the effects of an intermediate acting human insulin (NPH) and a long-acting insulin analog, insulin glargine, in insulin naïve type 2 diabetes patients, stratified by the type of hyperglycemia (fasting or postprandial type). Based on different action profiles, we hypothesized that patients having different hyperglycemia types would react differently when treated with these insulins. This is a post hoc analysis of the Lanmet study data. The Lanmet study was a randomized, 36-week controlled insulin initiation study in type 2 diabetes patients. 109 subjects with baseline HbA1c >8.0 % (64 mmol/mol) completed the study. The patients were divided into two groups according to fasting glucose (mmol/l)/HbA1c (%) ratio. Patients with a ratio ≥1.3 were defined as having fasting type and those with a ratio <1.3 as having postprandial type hyperglycemia. The main outcome measures were change in HbA1c and body weight, and final insulin dose. Independently of insulin type, compared to patients with postprandial type hyperglycemia, those with fasting type hyperglycemia had 2.1 kg/m^2^ greater initial BMI (*p* = 0.044), gained 2.0 kg more weight (*p* = 0.020, adjusted for baseline BMI *p* = 0.035), and had 36 % greater final insulin dose/kg (*p* = 0.001). With respect to hyperglycemia type, there was no difference between NPH and glargine in their effects on HbA1c. When starting bedtime insulin in type 2 diabetes patients, those with fasting type hyperglycemia are prone to greater weight gain. Hyperglycemia type does not help in identifying patients who would benefit specially from either NPH insulin or insulin glargine.

## Introduction

Some patients with type 2 diabetes have high fasting values and only moderately elevated postprandial values (fasting type hyperglycemia), while others have mainly high postprandial glucose concentrations (postprandial type hyperglycemia) [1]. This variation may be related to differences in the pathogenesis of type 2 diabetes between individual patients [2] and the severity of the metabolic disturbance [1]. We have previously examined insulin initiation in type 2 diabetes patients with respect to the hyperglycemia type and concluded that not all poorly controlled type 2 diabetes patients should automatically be treated with an oral agent and bedtime insulin, but patients with postprandial type hyperglycemia might benefit more from treatment with two daily NPH insulin injections [1]. At the time of the data collection of that study, long-acting insulin analogs were not available. Due to its long and steady duration of action, insulin glargine [4–6] has a better effect than NPH insulin on postprandial glucose concentrations [7, 8]. On the other hand, subjects with fasting type hyperglycemia might benefit relatively more from bedtime NPH insulin as its action is strong in the early morning hours. Although the difference in the time action profiles of glargine and NPH has been well established, there are no studies examining whether patients with fasting as compared to postprandial type hyperglycemia benefit more from NPH than glargine insulin.

The objective of this retrospective study was to compare the effect of human NPH insulin and a long-acting insulin analog glargine in insulin naïve type 2 diabetes patients, stratified by the type of hyperglycemia. We hypothesized that patients having fasting type hyperglycemia treated with NPH insulin or having postprandial type hyperglycemia treated with insulin glargine would have a greater improvement in HbA1c compared with patients having postprandial type hyperglycemia treated with NPH insulin or having fasting type hyperglycemia treated with insulin glargine. Moreover, we wanted to examine whether the hyperglycemia type is associated with insulin dose and weight change after insulin initiation.

## Methods

### Design of the study

The study was a post hoc analysis of the data collected in the Lanmet study [9]. The original study was performed in Finland and in the United Kingdom applying note for guidance CPMP/OCH/135/95. The ethics committees in all participating sites approved the study. Each patient gave an informed consent. In that study, 109 type 2 diabetes patients were randomized to use metformin with either glargine (60 patients) or NPH insulin (49 patients) as a bed-time injection. The sulphonylureas were stopped at randomization, but metformin was continued with unchanged dose throughout the study. According to the protocol, the insulin dose was aggressively raised until the target value of fasting plasma glucose (below 5.5 mmol/l) was achieved. In the present analysis, we divided the 109 type 2 diabetes patients that completed the study into two groups according to their hyperglycemia type. This was determined by the fasting plasma glucose/glycosylated hemoglobin ratio (mmol/l/%) as described previously [1]. Those who had a ratio over or equal to 1.3 (*N* = 57) formed the fasting type hyperglycemia group, and those whose ratio was less than 1.3 formed the postprandial type hyperglycemia group (*N* = 52).

### Subjects

The study subjects were type 2 diabetes patients treated with a stable dose (any dose) of sulphonylureas and metformin (≥1.5 g daily) or metformin alone for at least 3 months prior to screening. Their mean age was 56 years and diabetes duration 9 years. HbA1c was ≥8.0% and fasting plasma glucose ≥7.0 mmol/l. They were C-peptide positive (≥0.33 nmol/l) and insulin naïve. Patients with positive GAD antibodies, abnormal safety laboratory tests, history of alcohol or drug abuse, as well as those using other antihyperglycemic agents were not included. 109 patients were eligible to participate in the study that lasted for 36 weeks.

### Laboratory methods

HbA1c was measured by high-pressure liquid chromatography using the fully automated Glycosylated Hemoglobin Analyzer System (Bio-Rad, Richmond, CA, USA) traceable to the Diabetes Control and Complications Trial reference method, with a reference range of 4.0–6.0%. Serum concentrations of C-peptide and GAD antibodies were determined by RIA. S-ALT activity, high sensitivity CRP, and serum lipid and lipoproteins were determined by standard clinical laboratory methods.

### Outcome measures

Outcome measures were changes in fasting plasma glucose, HbA1c, and body weight as well as final insulin dose and occurrence of hypoglycemic events.

### Statistical analyses

Two-group comparisons were performed comparing groups with the two insulin types or comparing groups with the two types of hyperglycemia. Two-group comparisons were also performed comparing the effect of the two insulin preparations within the two hyperglycemia type groups (Järjestysmuutos). Four groups were formed according to hyperglycemia type and insulin preparation used. Due to statistically significant differences in the baseline HbA1c and BMI between these groups, comparisons were made also adjusting for these variables when appropriate. Statistical analyses were performed with the SAS program, version 14 (*t* test, paired *t* test, Mann–Whitney *U* test, ANOVA, ANOVA of repeated measurements or ANCOVA, when appropriate).

## Results

Baseline characteristics of the four groups formed according to hyperglycemia type (fasting type or postprandial type), and insulin type (NPH or glargine) are shown in Table 1. By definition, baseline fasting glucose values were higher in fasting type hyperglycemia groups. HbA1c values did not differ significantly between the groups. Average metformin dose was similar in all groups, and the proportion of patients having a history of sulphonylurea use was comparable between the groups.

**Table 1:** Baseline characteristics by hyperglycemia type and insulin preparation

	Fasting type hyperglycemia						Postprandial type hyperglycemia						*p* value^a^
	Glargine (*n* = 35)		NPH (*n* = 22)		Glargine + NPH (*n* = 57)		Glargine (*n* = 25)		NPH (*n* = 27)		Glargine + NPH (*n* = 52)		
	Mean	SD	Mean	SD	Mean	SD	Mean	SD	Mean	SD	Mean	SD	
Sex (%, M/F)	54/46		64/36		58/42		76/24		67/33		71/29		n.s.
Age (years)	55.9	8.7	57.0	7.7	58.3	8.3	56.6	10.6	57.9	9.2	57.3	9.8	n.s.
BMI (kg/m^2^)	32.1	4.7	33.5	6.3	32.7	5.4	30.1	5.8	30.7	4.2	30.6	5.0	0.044
Fasting plasma glucose (mmol/l)	13.8	2.4	14.1	1.8	13.9	2.2	10.6	1.7	10.6	1.6	10.6	1.7	<0.001
HbA1c (%)	9.0	1.2	9.4	1.0	9.1	1.1	9.2	1.0	9.2	1.1	9.2	1.0	n.s.
HbA1c (mmol/mol)	75	13	79	11	76	12	77	11	77	11	77	11	n.s.
fP-Gluc/HbA1c (mmol/l/%)	1.6	0.3	1.5	0.2	1.5	0.2	1.2	0.1	1.2	0.1	1.2	0.1	<0.001
Metformin dose (g)	2.3	0.5	2.2	0.4	2.2	0.4	2.3	0.4	2.1	0.3	2.2	0.4	n.s.
Sulphonylurea users (%)	77.1		86.4		80.7		80.8		85.2		82.7		n.s.
Hypertension (%)^b^	74.3		90.9		80.7		64.0		77.8		71.2		n.s.
fP-Chol (mmol/l)	5.07	1.96	4.91	0.90	5.00	1.08	4.73	0.93	5.10	0.92	4.92	0.94	n.s.
fP-HDL-Chol (mmol/l)	1.18	0.35	1.12	0.21	1.16	0.30	1.18	0.23	1.16	0.30	1.17	0.27	n.s.
fP-Trigly (mmol/l)^c^	2.47	1.28	2.73	1.21	2.57	1.25	2.12	1.48	2.31	1.95	2.22	1.73	0.018
hs-CRP (mg/l)^c^	4.8	5.9	3.3	3.6	4.2	5.2	2.53	3.4	2.16	2.4	2.3	2.9	0.010
S-ALT (IU/l)	50.6	38.8	46.0	36.3	48.9	37.7	34.6	19.7	35.6	17.0	35.1	18.2	0.019

^*a*^ *p* values show the significance of differences between combined groups (Glargine + NPH) in fasting and postprandial hyperglycemia types

^b^Antihypertensive medication or systolic blood pressure >140 mmHg or diastolic blood pressure >90 mmHg

^c^Statistical analysis after logarithmic transformation

Compared to patients with postprandial type hyperglycemia, those having fasting type hyperglycemia had significantly higher serum triglyceride, hs-CRP, and ALT concentrations. They also tended to have slightly more often hypertension (80.7 vs 71.2%), but this difference was not statistically significant.

**Fig. 1:**
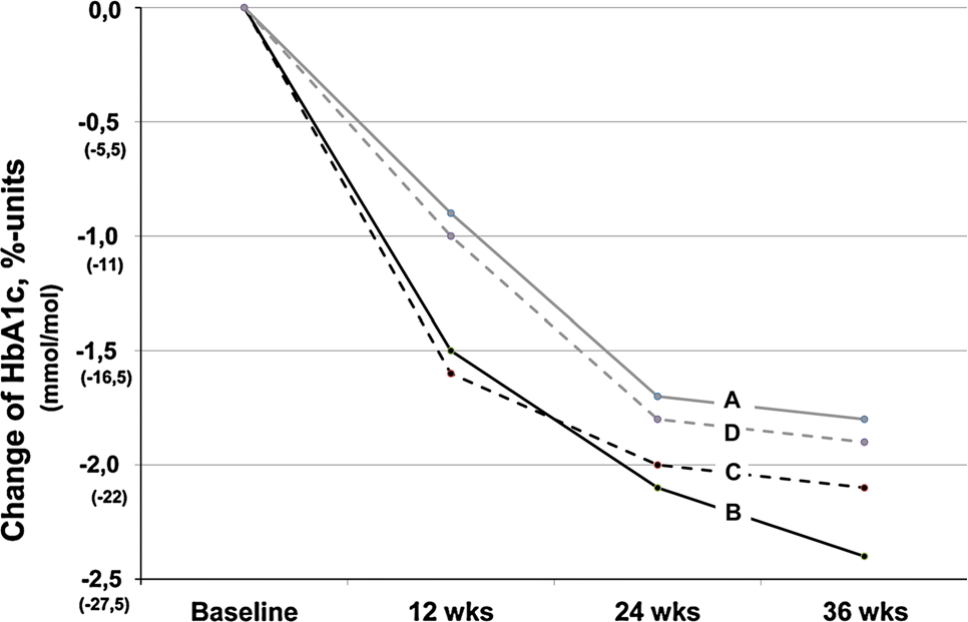
Decrease of HbA1c in patients with fasting or postprandial type hyperglycemia using glargine or NPH insulin. *A* Fasting type glargine, *B* fasting type NPH, *C* postprandial type glargine, *D* postprandial type NPH

In subjects using glargine, there was a statistically significantly greater (*p* = 0.034) decrease of HbA1c in postprandial type hyperglycemia group compared with that in the fasting type hyperglycemia group (Table 2; Fig. 1). This, however, was no more the case, when the groups were adjusted for baseline HbA1c (*p* = 0.489) or baseline BMI (*p* = 0.493). In subjects using NPH insulin, there was a tendency toward a greater decrease in HbA1c in fasting type hyperglycemia group compared with postprandial type hyperglycemia group (*p* = 0.075).

**Table 2:** Body weight, glucose control, insulin dose, and hypoglycemic events during the trial

	Fasting type hyperglycemia						Postprandial type hyperglycemia						*p* value^a^
	Glargine (*n* = 35)		NPH (*n* = 22)		Glargine + NPH (*n* = 57)		Glargine (*n* = 25)		NPH (*n* = 27)		Glargine + NPH (*n* = 52)		
	Mean	SD	Mean	SD	Mean	SD	Mean	SD	Mean	SD	Mean	SD	
Body weight (kg)													
0 week	94.0	18.1	98.6	22.2	95.8	19.7	90.8	19.5	90.9	13.6	90.9	15.9	n.s.
12 weeks	95.6	18.5	100.2	22.2	97.4	19.9	91.2	19.5	92.2	14.4	92.7	16.4	n.s.
24 weeks	96.7	18.5	102.3	24.1	98.9	20.8	91.8	20.1	92.6	15.1	92.2	16.9	0.069
36 weeks	97.5	19.3	103.5	24.4	99.8	21.4	92.3	18.8	93.3	15.1	92.8	16.8	0.064
Δ0 versus 36 weeks	3.4	4.9	4.9	5.0	4.0	4.9	1.5	3.4	2.4	4.3	2.0	3.9	0.020
Fasting blood glucose (mmol/l)													
0 week	13.8	2.4	14.1	1.8	13.9	2.2	10.7	1.8	10.6	1.5	10.6	1.7	<0.001
12 weeks	6.5	1.4	7.1	2.1	6.7	1.7	6.3	1.6	6.6	2.0	6.4	1.8	n.s.
24 weeks	6.2	1.7	6.7	2.2	6.4	1.9	6.6	1.5	6	1.3	6.3	1.4	n.s.
36 weeks	6.4	1.6	5.9	1.6	6.2	1.6	6.4	2.6	5.9	1.6	6.2	2.2	n.s.
Δ0 versus 36 weeks	−7.4	2.9	−8.2	2.2	−7.7	2.6	−4.2	2.8	−4.7	2.5	−4.5	2.7	<0.001
HbA1c (%)													
0 week	9.0	1.2	9.4	1.0	9.1	1.1	9.4	1.1	9.2	1.1	9.2	1.0	n.s.
12 weeks	8.1	1.2	7.9	1.1	8.0	1.1	7.8	1	8.2	1.0	7.9	0.9	n.s.
24 weeks	7.3	1.0	7.2	0.9	7.2	1.0	7.3	1	7.4	0.8	7.3	0.9	n.s.
36 weeks	7.2	0.9	6.9	0.8	7.1	0.9	7.1	1	7.3	1.1	7.2	1.0	n.s.
Δ0 versus 36 weeks	−1.8	1.1	−2.4	0.7	−2.1	1.0	−2.1	1.3	−1.9	1.3	−2.0	1.3	n.s.
HbA1c (mmol/mol)													
0 weeks	75	13	79	11	76	12	77	11	77	12	77	11	n.s.
12 weeks	65	13	63	12	64	12	60	9	65	10	63	10	n.s.
24 weeks	56	11	55	10	55	11	55	9	57	10	56	10	n.s.
36 weeks	55	10	52	9	54	10	55	11	57	12	55	11	n.s.
Δ0 versus 36 weeks	−20	13	−26	8	−23	11	−23	14	−20	14	−22	14	n.s.
Insulin dose (U/kg)													
36 weeks	0.77	0.40	0.78	0.26	0.78	0.36	0.56	0.29	0.58	0.30	0.57	0.25	0.001
Hypoglycemic events per patient													
0–12 weeks	0.6		1.4		1.0		1.2		2.6		2.0		0.055
13–24 weeks	0.9		1.4		1.1		1.4		2.1		1.8		0.100
25–36 weeks	1.2		1.8		1.5		2.2		1.7		1.9		n.s.
0–36 weeks	2.8		4.6		3.5		4.6		6.3		5.7		0.061

^a^ *p* values show the significance of differences between combined groups (Glargine + NPH) in fasting and postprandial hyperglycemia types

The combined group of patients having fasting type hyperglycemia and treated with NPH or postprandial type hyperglycemia treated with glargine had a greater improvement of glycemia compared to the combined group with fasting type hyperglycemia treated with glargine or postprandial type hyperglycemia treated with NPH (*p* = 0.046, after adjustment for baseline HbA1c *p* = 0.052). However, this difference disappeared after further adjustment for baseline BMI (*p* = 0.813).

Baseline BMI of the subjects having fasting type hyperglycemia was 2.1 kg/m^2^ greater than that of those having postprandial type hyperglycemia (*p* = 0.044). The weight gain of fasting type hyperglycemia patients during the study was 2.0 kg greater (*p* = 0.020; after adjustment for baseline BMI, *p* = 0.035) than of those, whose hyperglycemia was of postprandial type. The weight gain was not different between patients using NPH or glargine insulin.

Fasting plasma glucose decreased in all four groups. The decrease was smaller in both postprandial type hyperglycemia groups compared to fasting type hyperglycemia groups (*p* < 0.001). There was no difference (*p* = 0.667) in the decrease of fasting glucose between NPH and glargine using patients either within the fasting type or within the postprandial type group.

Independently of insulin type, the final insulin dose was on the average 0.77 IU/kg body weight in fasting type hyperglycemia group but only 0.57 IU/kg in patients with postprandial type hyperglycemia. This difference was statistically significant (*p* = 0.001).

During the first 3 months, NPH insulin treatment was associated with more hypoglycemic events than glargine (*p* = 0.05). This difference between insulin types was not associated with the type of hyperglycemia and disappeared with time. When both insulin types were combined, subjects with postprandial type hyperglycemia tended to have more hypoglycemias (*p* = 0.055). This difference also disappeared after the three first months of the study.

## Discussion

As far as we know, there is only one previous study on the initiation of insulin therapy in type 2 diabetes patients taking into account the type of hyperglycemia [1]. In that study, NPH insulin given twice daily resulted in postprandial type hyperglycemia (termed as “overall hyperglycemia” in that study) patients in a greater decrease in HbA1c compared with a combination of bedtime NPH + sulfonylurea or metformin. In fasting type hyperglycemia patients such an advantage of NPH insulin twice daily was not observed. The hypothesis of the present study was that the effects of a long-acting analog glargine and NPH insulin would be different in patients that have different hyperglycemia types. In line with the hypothesis, in unadjusted analysis, a combined group of patients having fasting type hyperglycemia and treated with NPH or postprandial type hyperglycemia treated with glargine had a greater improvement of glycemia compared to a combined group of patients with fasting type hyperglycemia treated with glargine or postprandial type hyperglycemia treated with NPH (*p* = 0.046). However, after adjustment for baseline HbA1c and BMI, there was no more such a difference. Thus, the two insulin types seem not to differ in their effect on HbA1c decline with respect to the type of hyperglycemia.

**Fig. 2:**
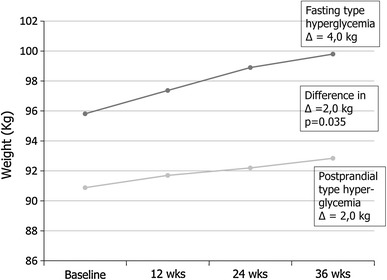
Increase of body weight in patients with fasting type or postprandial type hyperglycemia. Patients using NPH or glargine insulin are combined in the two hyperglycemia type groups

Initiation of insulin therapy is known to cause an unwanted increase of BMI [10]. We found that hyperglycemia type was significantly associated with adiposity: those who had fasting type hyperglycemia were significantly more obese than those with postprandial type hyperglycemia. Those with fasting hyperglycemia also gained 2.0 kg more weight after insulin initiation (Fig. 2; *p* = 0.020, after adjustment for baseline BMI, *p* = 0.035). To achieve a similar glycemic control, they also needed significantly more insulin.

Compared to patients with postprandial type hyperglycemia, patients with fasting type hyperglycemia not only had significantly higher baseline BMI, but they also had higher serum triglyceride levels and a non-significant tendency toward higher prevalence of hypertension, i.e., two components of the metabolic or insulin resistance syndrome [10] In accordance with this, fasting type hyperglycemia patients also exhibited greater degree of low-grade inflammation (higher hs-CRP values) known to be associated with insulin resistance [11]. Moreover, fasting type hyperglycemia patients had higher serum ALT concentration reflecting higher liver fat content known to be connected with insulin resistance [12]. Finally, the higher insulin dose needed by these patients supports the idea that fasting type hyperglycemia patients are more insulin resistant than postprandial type hyperglycemia patients.

It would be interesting to study whether similar associations between hyperglycemia type and increase in body weight are present when starting insulin treatment with insulin detemir as previous studies have suggested that it may cause less weight gain than NPH or glargine insulin after insulin initiation [13–15].

We determined the hyperglycemia type by the fasting plasma glucose/HbA1c ratio as described previously [1] Patients with a higher ratio (≥1.3) were defined as having fasting type hyperglycemia, whereas those, whose ratio was below 1.3, were defined as having postprandial type hyperglycemia. The value 1.3 was calculated based on the former diagnostic fasting plasma glucose value 7.8 mmol/l [16] and upper normal limit of HbA1c 6.0%. One may argue the use of some other fasting plasma glucose/HbA1c ratio to distinguish fasting type and postprandial type hyperglycemia patients. However, the ratio 1.3 resulted in fasting type and postprandial type hyperglycemia groups of approximately same size in the present study as it did also in the previous study [1], allowing relevant statistical comparisons.

The strength of the present study is that it was based on a controlled randomized trial, the Lanmet study [9]. Its limitation was that the number of patients in the four subgroups is relatively small. The findings on the associations between hyperglycemia type and adiposity and weight gain should therefore be confirmed in a study with a greater number of diabetes patients.

The main message of this study is that the patient’s glucose profile predicts risk of weight gain, which is a known problem in starting insulin treatment in type 2 diabetes patients [17, 18]. This profile can be easily calculated from the fasting glucose/HbA1c ratio. Analog insulin cannot automatically be held preferable to the less expensive NPH insulin [19].

## References

[1] Vähätalo M, Rönnemaa T, Viikari J (2007). Recognition of fasting or overall hyperglycaemia when starting insulin treatment in patients with type 2 diabetes in general practice. Scand J Prim Health Care.

[2] Stančáková A, Javorský M, Kuulasmaa T, Haffner SM, Kuusisto J, Laakso M (2009). Changes in insulin sensitivity and insulin release in relation to glycemia and glucose tolerance in 6,414 Finnish men. Diabetes.

[3] Monnier L, Lapinski H, Colette C (2003). Contributions of fasting and postprandial plasma glucose increments to the overall diurnal hyperglycemia of type 2 diabetic patients: variations with increasing levels of HbA(1c). Diabetes Care.

[4] Bazzano LA, Lee LJ, Shi L, Reynolds K, Jackson JA, Fonseca V (2008). Safety and efficacy of glargine compared with NPH insulin for the treatment of Type 2 diabetes: a meta-analysis of randomized controlled trials. Diabet Med.

[5] Bradley C, Gilbride CJ (2008). Improving treatment satisfaction and other patient-reported outcomes in people with type 2 diabetes: the role of once-daily insulin glargine. Diabetes Obes Metab.

[6] Massi Benedetti M, Humburg E, Dressler A, Ziemen M (2003). A 1-year, randomised, multicentre trial comparing insulin glargine with NPH insulin in combination with oral agents in patients with type 2 diabetes. Horm Metab Res.

[7] HOE 901/3002 Study Group, Yki-Järvinen H, Dressler A, Ziemen M, HOE 901/300 s Study Group (2000) Less nocturnal hypoglycemia and better post-dinner glucose control with bedtime insulin glargine compared with bedtime NPH insulin during insulin combination therapy in type 2 diabetes. Diabetes Care 23(8):1130–1136 10.2337/diacare.23.8.1130 10937510

[8] Lee P, Chang A, Blaum C, Vlajnic A, Gao L, Halter J (2012). Comparison of safety and efficacy of insulin glargine and neutral protamine hagedorn insulin in older adults with type 2 diabetes mellitus: results from a pooled analysis. J Am Geriatr Soc.

[9] Yki-Järvinen H, Kauppinen-Mäkelin R, Tiikkainen M, Vähätalo M, Virtamo H, Nikkilä K, Tulokas T, Hulme S, Hardy K, McNulty S, Hänninen J, Levänen H, Lahdenperä S, Lehtonen R, Ryysy L (2006). Insulin glargine or NPH combined with metformin in type 2 diabetes: the LANMET study. Diabetologia.

[10] DeFronzo RA, Ferrannini E (1991). Insulin resistance. A multifaceted syndrome responsible for NIDDM, obesity, hypertension, dyslipidemia, and atherosclerotic cardiovascular disease. Diabetes Care.

[11] Shoelson SE, Herrero L, Naaz A (2007). Obesity, inflammation, and insulin resistance. Gastroenterology.

[12] Gómez-Sámano MA, Cuevas-Ramos D, Mehta R, Brau-Figueroa H, Meza-Arana CE, Gulias-Herrero A (2012). Association of alanine aminotransferase levels (ALT) with the Hepatic Insulin Resistance Index (HIRI): a cross-sectional study. BMC Endocr Disord.

[13] Baxter MA (2008). The role of new basal insulin analogues in the initiation and optimization of insulin therapy in type 2 diabetes. Acta Diabetol.

[14] Monami M, Marchionni N, Mannucci E (2008). Long-acting insulin analogues versus NPH human insulin in type 2 diabetes: a meta-analysis. Diabetes Res Clin Pract.

[15] Owen V, Seetho I, Idris I (2010). Predictors of responders to insulin therapy at 1 year among adults with type 2 diabetes. Diabetes Obes Metab.

[16] Diabetes mellitus (1985) Report of a WHO Study Group. World Health Organization technical report series 727. Geneva: World Health Organization 3934850

[17] Pontiroli AE, Miele L, Morabito A (2011). Increase of body weight during the first year of intensive insulin treatment in type 2 diabetes: systematic review and meta-analysis. Diabetes Obes Metab.

[18] McAdam-Marx C, Bouchard J, Aagren M, Nelson R, Brixner D (2010). Analysis of glycaemic control and weight change in patients initiated with human or analog insulin in an US ambulatory care setting. Diabetes Obes Metab.

[19] Levin P (2008). The cost-effectiveness of insulin glargine versus neutral protamine hagedorn insulin in type 2 diabetes: a focus on health economics. Diabetes Obes Metab.

